# MBMSA-UNet: A Multi-Scale Attention-Based Instance Segmentation Model for Moso Bamboo Cells

**DOI:** 10.3390/plants15060969

**Published:** 2026-03-20

**Authors:** Xue Zhou, Ziwei Cheng, Long Chen, Jiawei Pei, Yingyu Liao, Weizhang Liu, Chunyin Wu, Changyu Liu

**Affiliations:** 1 College of Mathematics and Informatics, South China Agricultural University, Guangzhou 510642, China; zys212121@gmail.com (X.Z.); chengzw@nfu.edu.cn (Z.C.); amazarashi.sdk@gmail.com (L.C.); cavey759@gmail.com (J.P.); liaoyingyu18@163.com (Y.L.); liuweizhang@scau.edu.cn (W.L.); 2 Business School, Nanfang College Guangzhou, Guangzhou 510970, China; 3 School of Economics and Trade, Changzhou Technical Institute of Tourism & Commerce, Changzhou 213032, China

**Keywords:** moso bamboo cells, instance segmentation, U-Net, multi-scale attention, microscopic image analysis, vascular bundles, parenchyma cells

## Abstract

Instance segmentation of moso bamboo cells is a critical step in quantitative structural analysis of bamboo materials and plant phenomics research. Moso bamboo tissues are mainly composed of vascular bundles and parenchyma cells. Within vascular bundles, fiber cells exhibit thick cell walls and extremely dense arrangements, whereas vessel cells are characterized by large diameters and complex internal structures. These features frequently lead to blurred boundaries, structural complexity, and local overexposure in microscopic images, making it difficult for traditional segmentation algorithms to achieve stable and accurate results. Although the U-Net has demonstrated outstanding performance in biological microscopic image analysis, its feature extraction capability and boundary recognition stability remain insufficient when dealing with the composite structure of moso bamboo. To address these challenges, this study proposes an improved model based on a multi-scale attention mechanism, termed MBMSA-UNet (Moso Bamboo Multi-Scale Attention U-Net). Building upon the encoder–decoder architecture of U-Net, the proposed model introduces a multi-scale channel-spatial attention block, aiming to handle the pronounced morphological and scale differences among vessels, fibers, and parenchyma cells. By adaptively reweighting features at different scales, the model enhances cross-layer feature fusion and strengthens responses to key regions, thereby effectively suppressing local overexposure interference and emphasizing boundary features between different cell types. Experimental results demonstrate that, compared with the U-Net and several of its improved variants, MBMSA-UNet achieves higher segmentation accuracy and greater robustness on microscopic images of moso bamboo, providing a solid foundation for fine-grained quantitative analysis of complex bamboo tissues.

## 1. Introduction

Instance segmentation of moso bamboo (*Phyllostachys edulis*) cells is a fundamental task for investigating bamboo growth mechanisms and the mechanical properties of bamboo materials. As a typical fast-growing plant with high carbon sequestration efficiency, the cellular structure of moso bamboo underlies its excellent mechanical and ecological performance [1,2,3,4]. At the microscopic level, bamboo tissues are primarily composed of vascular bundles and parenchyma cells, which exhibit diverse morphologies and relatively weak boundaries [1,5]. Moso bamboo plays a critical role in global environmental protection, achieving carbon neutrality, and ecological restoration goals [6,7,8,9]. However, large-scale harvesting requires a deeper understanding of bamboo cellular structures and growth patterns [10,11,12]. Cellular structures vary significantly across different internode positions, which directly affects structural analysis [13,14]. To reveal these characteristics, various microscopic imaging techniques have been employed, including optical microscopy [1], X-ray microscopy [15,16], micro-CT [17,18], time-lapse microscopy [19], and transmission electron microscopy [20]. These studies have successfully elucidated the spatiotemporal evolution of cell types during bamboo development [21,22]. By enabling the automatic identification of individual cells, researchers can quantitatively analyze cell morphology and spatial arrangement, providing precise data support for mechanical and growth modeling. Vascular bundles serve as functional units integrating transport and mechanical support, consisting mainly of two key cell types. First, vessel cells function as conducting tissues responsible for longitudinal transport; morphologically, they appear as large-lumen structures often accompanied by lignified and thickened cell walls. Second, fiber cells are mainly distributed around the vascular bundles to form a mechanical sheath; they are characterized by elongated shapes, extremely thick walls, and exceptionally dense arrangements. This complex cellular composition poses significant challenges for automated instance segmentation. The dense arrangement of fiber cells results in highly ambiguous intercellular boundaries, while the large size of vessel cells and the irregular morphology of parenchyma cells introduce pronounced scale variations. Furthermore, local overexposure and low signal-to-noise ratios frequently occur during image acquisition, further increasing the difficulty of precise segmentation.

Constructing comprehensive three-dimensional structural models requires high-precision algorithms capable of accurately distinguishing cell types and extracting precise boundaries. Existing image segmentation methods fall into traditional image processing approaches and deep learning-based methods. Traditional approaches rely on handcrafted features but show limited performance on dense bamboo microstructures, often leading to under-segmentation or misclassifying overexposed textures as noise. Furthermore, they are highly sensitive to illumination and imaging conditions [23]. Convolutional neural networks (CNNs) have provided new solutions through end-to-end feature learning, effectively extracting multi-scale features and suppressing pseudo-texture interference. Nevertheless, standard convolution kernels often struggle to capture features across the extreme scale discrepancies between small fiber cells and large vessel cells [24,25]. In the field of general cell segmentation, models like Cellpose [26] and HoVer-Net [27] achieve unified segmentation and fine-grained boundary extraction. In plant science, deep learning is widely applied. For instance, PlantSeg [28] and methods developed by Smith et al. [29] provide robust cell-level segmentation. Recent reviews also show notable progress in crop disease detection and plant phenotyping [30,31]. Among deep learning models, U-Net and its variants are highly representative in biological microscopic image analysis [32,33,34]. However, when applied to moso bamboo, the standard U-Net suffers from boundary information loss, false responses to internal overexposure, and difficulty handling the strong adhesion and scale disparity of dense vascular bundles [35]. To address these issues, numerous improvements have been proposed, such as RSU-Net [36], MSECA-U-Net [37], ECA-Net integrations [38], E-Res U-Net [39], and SDAU-Net [40]. Other variants have utilized SE modules [32], densely connected blocks [33], or optimized information fusion during upsampling [34]. Although promising, these methods still struggle to simultaneously achieve fine-grained boundary delineation and effective multi-scale modeling for complex bamboo tissues.

To address the key bottlenecks of blurred boundaries, local overexposure, and significant structural scale variations, this study proposes an improved instance segmentation model tailored for moso bamboo cells, namely MBMSA-UNet (Moso Bamboo Multi-Scale Attention U-Net). Built upon the classical U-Net framework, the proposed model introduces a multi-scale channel-spatial attention module to adaptively adjust feature weights, enhance boundary responses, and effectively suppress internal overexposure. The network predicts both cell regions and boundary-related features, enabling reliable instance-level segmentation of densely packed cells during post-processing. Experimental results demonstrate that MBMSA-UNet significantly outperforms conventional U-Net and its improved variants [36,37,38,39,40], achieving clear advantages in segmentation accuracy and boundary integrity. This approach provides a new, robust solution for the automated segmentation of complex plant cells and establishes a methodological foundation for future studies on bamboo growth mechanisms and tissue structural analysis [41].

## 2. Materials and Methods

### 2.1. MBVB-PaC Dataset

#### 2.1.1. Data Acquisition and Labeling

**Figure 1:**
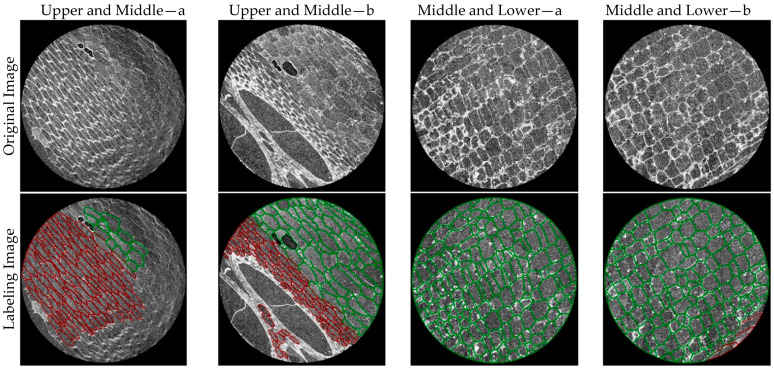
Representative annotated samples from the MBVB-PaC dataset. The red color indicates vascular bundles, and the green color indicates parenchyma cells.

To systematically study the three-dimensional microstructural characteristics of moso bamboo culm tissue, we constructed a micro-CT image dataset containing vascular bundles and pith cells, named MBVB-PaC (Moso Bamboo Vascular Bundle and Pith Cell Dataset) (Figure 1). The growth pattern of moso bamboo cells is such that their height and morphology are essentially completed within a single growing season, after which they no longer grow taller but only undergo tissue maturation and cell wall thickening. The moso bamboo samples used in this study were collected from moso bamboo plants in Zhejiang Province, China, which is a high-yielding region for moso bamboo with a warm and humid climate and abundant rainfall, suitable for moso bamboo growth. In this region, the moso bamboo takes approximately 40 to 60 days to mature. Therefore, samples were collected from moso bamboo plants after the formation stage to facilitate understanding of the cell growth pattern after this stage. The samples were collected from the upper and middle parts of individual moso bamboo culms. The extracted tiny samples had a physical size of 0.0503 cm × 0.0518 cm × 0.1905 cm and were scanned using a micro-CT system to obtain continuous cross-sectional slices with a resolution of 1939 × 1995 pixels. The ratio of the size of moso bamboo cells in the slice image to the actual moso bamboo cells is 1:0.2956. A total of 1400 images were collected, including 800 from the upper and middle parts and 600 from the lower part. Compared to traditional microscopic observation methods, CT scanning technology can non-destructively acquire high-resolution image information. More and more researchers are applying CT technology to plant cell research. The spatial resolution of micro-CT can reach the micrometer or even submicrometer level, allowing for clear observation of plant cell structures through the images obtained. By adopting this high-precision, fine-step scanning strategy, key structural features of moso bamboo tissue, including the thick walls and dense arrangement of fiber cells, the significant lumens of vessel cells, and the overall structural complexity of vascular bundles, were comprehensively captured. Since the scanning step length is much smaller than the typical cell diameter, individual cells repeatedly appear in multiple continuous slices, ensuring complete recording of information from different cross-sections. This redundancy enables the dataset to retain richer three-dimensional structural details.

The acquired images were initially pre-labeled using the Cellpose cell segmentation model to automatically generate label files. Each cell instance was assigned an individual label, and the corresponding boundary coordinates were recorded in text format, where straight-line connections between successive coordinate points formed closed cell contours. This pre-annotation strategy substantially reduced the manual labeling workload. The generated text-based labels were subsequently converted into JSON format, and the cell categories were manually refined in LabelMe 5.2.1 by relabeling instances as either vascular bundle or parenchyma cell, resulting in a two-class annotated dataset.

Based on the annotation results, the original microscopic CT images were organized into paired image-mask samples, where input images were represented as three-channel RGB images, and corresponding masks were single-channel integer-labeled maps. The dataset included three categories: background, parenchyma cells, and vascular bundles.

#### 2.1.2. Data Augmentation

**Figure 2:**
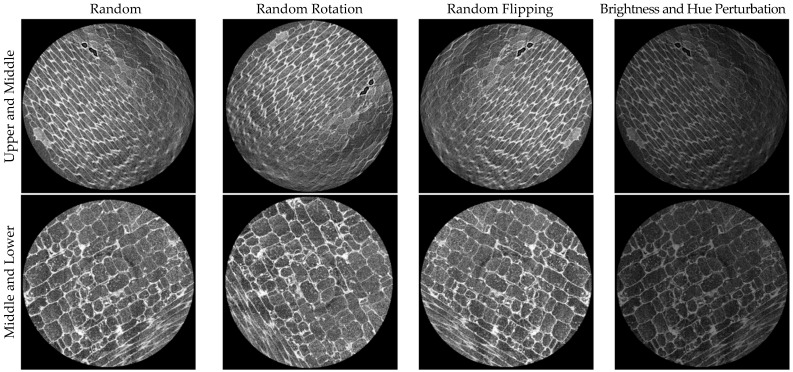
Samples of the applied data augmentation strategies.

Before dataset splitting, each original image was evenly partitioned into nine non-overlapping patches in a 3 × 3 layout. This strategy reduced information waste caused by direct cropping, preserved more useful local details, and effectively expanded the dataset beyond the original 1400 images to 12,600 images. The dataset was then randomly split into training and validation subsets at an 8:2 ratio, with category distributions kept as balanced as possible across the two subsets. To reduce the potential bias caused by a single train–validation split, a 5-fold cross-validation experiment was conducted, and the average performance is reported. Prior to network input, the original single-channel discrete masks were converted into three-channel one-hot encoded representations to support multi-class pixel-wise classification loss functions and evaluation metrics. Subsequently, a series of synchronized data augmentation operations was applied to image–mask pairs to enhance model generalization and alleviate overfitting, which were as follows: (1) Random Rotation: For each sample, a rotation angle was randomly sampled with a probability of 25%, and both the image and its corresponding mask were rotated using the same affine transformation. Nearest-neighbor interpolation was employed to prevent label ambiguity or boundary blurring in the masks. (2) Random Flipping: For each sample, a random number drawn from a uniform distribution *U*(0, 1) was used to determine conditional flipping operations. Vertical flipping was applied when the random value was less than 0.25, while horizontal flipping was applied when the value exceeded 0.75. This strategy resulted in approximately 25% probability for both vertical and horizontal flips, effectively simulating variations in slicing orientation and imaging direction. (3) Brightness and Hue Perturbation: During training, random brightness and hue augmentations were applied exclusively to the input images. The maximum brightness variation was set to 0.1, and the maximum hue variation was set to 0.5. Pixel values were clipped to the range [0, 1] after augmentation (Figure 2). These operations were not applied to the masks, ensuring structural consistency while increasing diversity in illumination conditions and staining variations.

**Figure 3:**
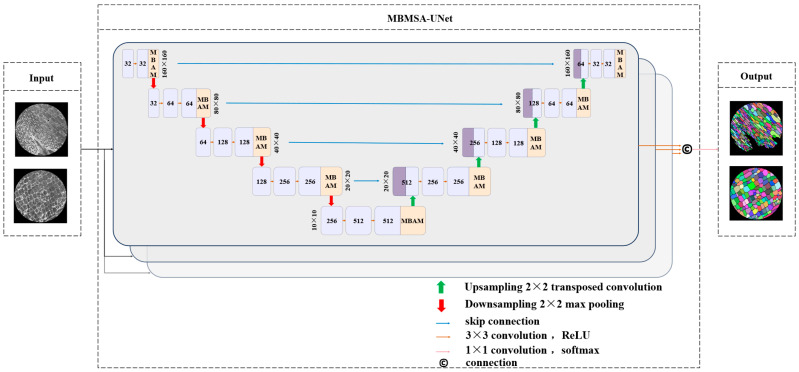
Overall architecture of the MBMSA-UNet model.

Each MBVB-PaC image typically contains a variable number of parenchyma cells (approximately 100–300 per image), along with vascular bundle structures composed of vessels, fibers, and phloem tissues, providing a realistic and comprehensive sample basis for cellular morphology analysis. As shown in the annotated samples in Figure 1, the microscopic CT images exhibit characteristic features such as densely packed fiber regions, large vessel lumens, considerable background noise, blurred boundaries, and mixed cellular morphologies. These complex patterns reflect the natural anatomical organization of moso bamboo tissues and pose significant challenges for segmentation models, particularly in handling extreme scale variations and tightly adhered boundaries. Consequently, the MBVB-PaC dataset holds substantial academic and practical value for evaluating advanced cell segmentation methods (Figure 3).

### 2.2. MBMSA-UNet

#### 2.2.1. Architecture

The overall architecture of MBMSA-UNet is inspired by the triple-symmetric encoder–decoder design of UNet-ID (Figure 3). The network consists of three structurally identical but parameter-independent encoder–decoder branches, which operate in parallel to extract features and perform hierarchical semantic analysis on the input microscopic images. This triple-branch design enables the model to capture fine-grained differences in moso bamboo cell structures from multiple perspectives, scales, and representation pathways, thereby significantly enhancing sensitivity to subtle cell boundaries, textures, and low-contrast regions.

Within each branch, the encoder progressively extracts multi-scale features ranging from shallow texture information to high-level semantic representations through successive convolutional layers, downsampling operations, and feature aggregation. The decoder restores spatial resolution via upsampling and fuses shallow detail features with deep semantic features through skip connections, ensuring precise discrimination between cellular structures and background regions. The network predicts pixel-wise probability maps representing cellular regions in the microscopic images.

**Figure 4:**
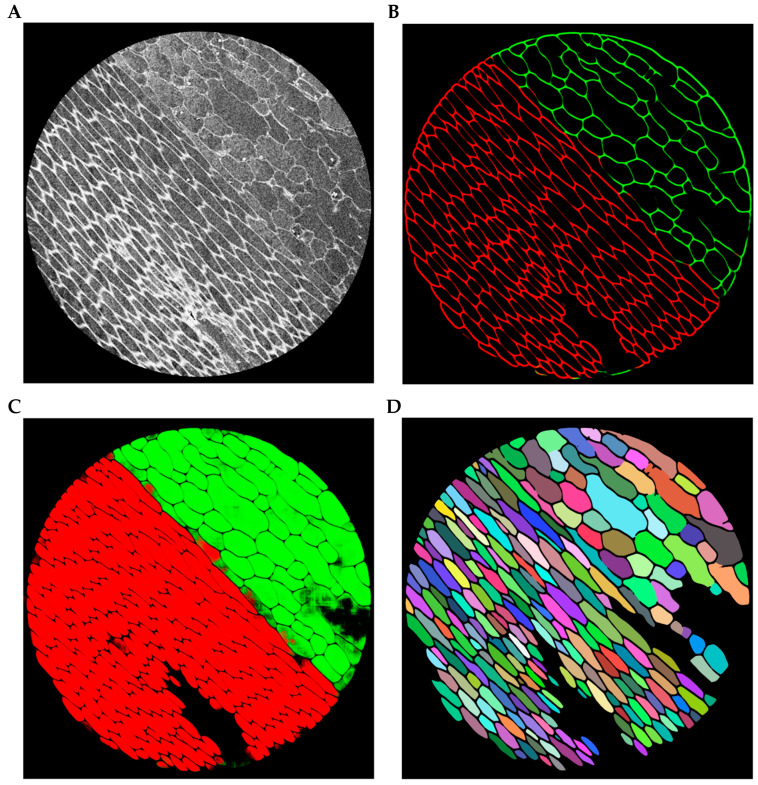
The boundary-guided instance generation process in MBMSA-UNet. (**A**) Original image. (**B**) Predicted boundaries, where vascular bundles are shown in red and parenchyma cells are shown in green. (**C**) Cellular interior regions, where vascular bundles are shown in red and parenchyma cells are shown in green. (**D**) Cell instances, where different colors represent different cell instances.

In moso bamboo tissues, neighboring cells are usually densely packed, and the boundaries between adjacent cells are often weak or partially indistinguishable. To alleviate this difficulty, the network is designed to enhance the representation of cell boundary features during feature extraction and decoding. Stronger boundary responses help highlight the cell walls that separate neighboring cells. During inference, the predicted probability maps are first converted into discrete segmentation masks. The enhanced boundary responses facilitate the separation of densely adhered cell regions. Subsequently, connected-component analysis is applied to identify individual connected regions, and each region is assigned a unique label to form a cell instance. In this way, instance-level segmentation of bamboo cells can be obtained while maintaining the stability of semantic segmentation training. The instance generation process is illustrated in Figure 4. Starting from the original micro-CT image (Figure 4A), the network predicts strong boundary responses that highlight the cell walls separating neighboring cells (Figure 4B). Based on these boundary cues, densely adhered cellular regions are separated to obtain individual cell interiors (Figure 4C). Finally, connected-component labeling assigns a unique identifier to each separated region, producing the final cell instances (Figure 4D).

To further strengthen feature representation, MBAM (Moso Bamboo Attention Module) blocks are introduced at two critical stages. In the first encoder stage, MBAM modules are inserted after convolutional blocks at each scale and before feature downsampling, enabling adaptive recalibration of channel-wise features to emphasize informative texture regions while suppressing redundant background noise. In the second decoder stage, MBAM modules are applied after each upsampling and skip-connection fusion step to mitigate noise amplification from shallow features and to enhance boundary representation consistency, thereby improving localization accuracy in complex boundary regions. This insertion strategy reinforces hierarchical feature propagation during encoding and suppresses mis-segmentation caused by local overexposure during decoding. In alignment with the challenges discussed in the Introduction, the proposed architecture specifically addresses blurred intercellular boundaries, local overexposure, and pronounced structural scale variations in moso bamboo microscopic images.

Let the input image be $x\in R^{H\times W\times C}$, and the network output be a pixel-wise foreground probability map $\hat{y}=\sigma (f_{\theta }(x)),$ where $f_{\theta }(\cdot )$ denotes the learnable mapping and σ(·) is the Sigmoid function. During training, a weighted combination of binary cross-entropy (BCE) loss and *Dice* loss, which could be denoted as $L=\lambda L_{BCE}+(1-\lambda )L_{Dice}$, is employed. For the validation set, only essential type conversions and one-hot encoding are performed, with no random augmentation applied, ensuring a stable data distribution and objective evaluation of model generalization performance.

#### 2.2.2. MBAM

**Figure 5:**
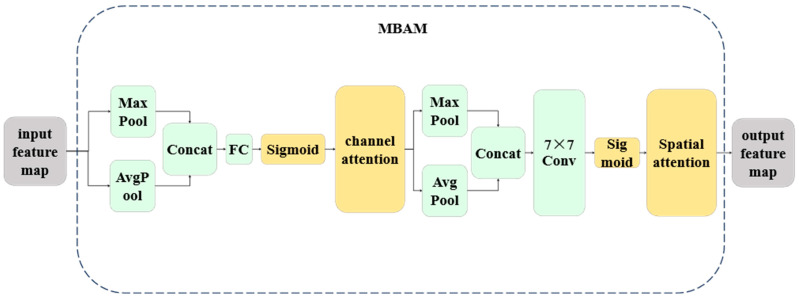
Architecture of the MBAM module.

To address the three major challenges of moso bamboo microscopic images—blurred intercellular boundaries, local overexposure, and significant structural scale variation—we designed the MBAM module (Figure 5). MBAM adopts a cascaded structure of channel recalibration followed by spatial saliency enhancement, enabling adaptive feature weighting during both downward feature propagation and skip-connection fusion to strengthen responses to critical cellular regions. Let the input feature map be $F\in R^{H^{\prime }\times W^{\prime }\times C}$. Channel Attention first adopts global average pooling to obtain a channel descriptor $z\in R^{C}.$ This descriptor is then passed through a two-layer fully connected bottleneck structure to generate a channel weight vector $S\in (0,\;1{)}^{C}$, where $W_{1}\in R^{\frac{C}{r}\times C}$, $W_{2}\in R^{C\times \frac{C}{r}}$, r denotes the channel reduction ratio, δ(·) is the ReLU activation, and σ(·) is the Sigmoid function. The channel-recalibrated feature map is denoted as $F_{i,\;j,\;c}^{\mathrm{CA}}=s_{c}\cdot F_{i,\;c,\;j}$. Spatial attention is then applied by performing average pooling and max pooling along the channel dimension of $F^{\mathrm{CA}}$, yielding two spatial maps $A,\;M\in R^{H^{\prime }\times W^{\prime }\times 1}.$ These maps are concatenated along the channel dimension and passed through a 7×7 convolution followed by a Sigmoid function to produce a spatial attention map $P\in (0,\;1{)}^{H^{\prime }\times W^{\prime }\times 1}.$

By applying channel recalibration prior to spatial gating, MBAM demonstrates more stable enhancement of boundary regions and low-contrast areas compared with alternative designs (e.g., spatial-first or parallel attention), while effectively suppressing erroneous activation of background textures in cell segmentation tasks. The parameter and computational overhead of MBAM are relatively small compared with the convolutional backbone. Given channel number C and reduction ratio r, the channel attention module introduces approximately $2C^{2}/r$ parameters, while the spatial attention module adds only 7×7×2=98 parameters. This overhead is negligible compared with standard convolution operations (e.g., two 3×3 convolutions with $9C_{in}C_{out}$ parameters). Under identical channel configurations and input resolutions, MBAM significantly improves boundary separation and weak-edge localization with acceptable computational cost. Inserting MBAM at the end of convolutional blocks allows global feature recalibration before downward propagation, reducing the accumulation of irrelevant responses. Placement after upsampling and skip fusion suppresses amplification of shallow textures during decoding while highlighting regions consistent with high-level semantics. To maintain training stability, MBAM is designed as a plug-and-play module decoupled from the backbone, without modifying the optimizer, learning rate schedule, loss function, or threshold settings. This design facilitates rapid reuse, fair comparison with existing U-Net variants, and transfer evaluation across different resolutions and microscopic imaging modalities. Compared with traditional attention modules, MBAM exhibits the following advantages: Its parameter design is tailored to the feature distribution characteristics of moso bamboo cell images, enabling simultaneous perception of morphologically distinct vascular bundle components (e.g., slender dense fibers and large-lumen vessels) and irregular parenchyma cells; it enhances interlayer semantic propagation and suppresses local overexposure during encoding while reinforcing boundary responses during decoding, yielding stable performance under low-contrast and high-blur conditions; and it maintains plug-and-play compatibility and optimization decoupling, allowing seamless integration into other bamboo cell analysis frameworks. With minimal additional parameters and high computational efficiency, MBAM consistently enhances blurred boundaries and overexposed regions in both middle-upper and middle-lower bamboo tissue samples, significantly improving the robustness and generalization of moso bamboo cell instance segmentation.

### 2.3. Experimental Settings

#### 2.3.1. Experimental Environment and Training Configuration

All experiments were conducted in identical hardware and software environments. Model construction and training were implemented through joint invocation of the R programming language and deep learning frameworks, with GPU acceleration enabled via CUDA. The main experimental environment configuration is summarized in Table 1.

All models were trained under comparable settings, while minor adjustments were made according to the recommended configurations in the original papers. The key training hyperparameters of our model are listed in Table 2.

A relatively small initial learning rate combined with an extremely low decay term facilitates stable convergence of the multi-branch attention-based U-Net architecture. The batch size of 12 balances gradient estimation stability and training efficiency.

#### 2.3.2. Evaluation Metrics

During training and validation, two metrics were recorded: the total loss and the *Dice* coefficient. The final model performance was evaluated using four metrics: *Dice*, *IoU*, *Overall Accuracy*, and ${mAP}_{50-95}^{mask}$. Let an image contain N pixels, with ground-truth mask $y=\{y_{i}{\}}_{i=1}^{N}\left(y_{i}\in \left\{0,\;1\right\}\right)$ and predicted mask $\hat{y}=\{{\hat{y}}_{i}{\}}_{i=1}^{N}$, where ${\hat{y}}_{i}\in [0,\;1]$ or ${\hat{y}}_{i}\in \{0,\;1\}$ after thresholding. A small constant ε>0 is added to avoid division by zero.

*Dice* (Sørensen–Dice coefficient) measures overlap quality between prediction and ground truth and is robust to class imbalance, making it particularly suitable for cell segmentation. The metric of *Dice* is defined as

(1) $$Dice=\left(2\sum_{i=1}^{N}y_{i}{\hat{y}}_{i}+\varepsilon \right)/\left(\sum_{i=1}^{N}y_{i}+\sum_{i=1}^{N}{\hat{y}}_{i}+\varepsilon \right)=\frac{2TP}{2TP+FP+FN+\varepsilon }$$

*IoU* (Intersection over Union) is more sensitive to boundary errors and reflects over-segmentation and under-segmentation effects. The metric of *IoU* is defined as

(2) $$IoU=\frac{\sum_{i=1}^{N}min(y_{i},\;{\hat{y}}_{i})}{\sum_{i=1}^{N}max(y_{i},\;{\hat{y}}_{i})+\varepsilon }=\frac{TP}{TP+FP+FN+\varepsilon }$$

*Overall Accuracy* reflects global pixel classification accuracy but may be overly optimistic when foreground occupies a small fraction of the image; thus, it is reported alongside *Dice* and *IoU*. The metric of *Overall Accuracy* is defined as

(3) $$Overall\;Accuracy=\frac{1}{N}\sum_{i=1}^{N}1[{\hat{y}}_{i}=y_{i}]=\frac{TP+TN}{N}$$

${mAP}_{50-95}^{mask}$ (mean Average Precision for masks) evaluates instance-level detection and localization quality across both lenient and strict overlap thresholds and is particularly sensitive to boundary precision and instance discrimination performance at higher thresholds (≥0.75). Predicted instance masks are matched to ground-truth masks in descending order of confidence using maximum *IoU* or Hungarian matching. For *IoU* thresholds t∈{ 0.50, 0.55, …, 0.95 }, the average precision ${AP}^{mask}(t)$ is computed as the area under the precision–recall curve. The metric of ${mAP}_{50-95}^{mask}$ is defined as

(4) $${mAP}_{50-95}^{mask}=\frac{1}{10}\sum_{t\;\in \;\{\;0.50,\;0.55,\;\ldots ,\;0.95\;\}}{AP}^{mask}(t)$$

## 3. Results

### 3.1. Comparative Experiments

#### 3.1.1. Comparison of Attention Modules

**Figure 6:**
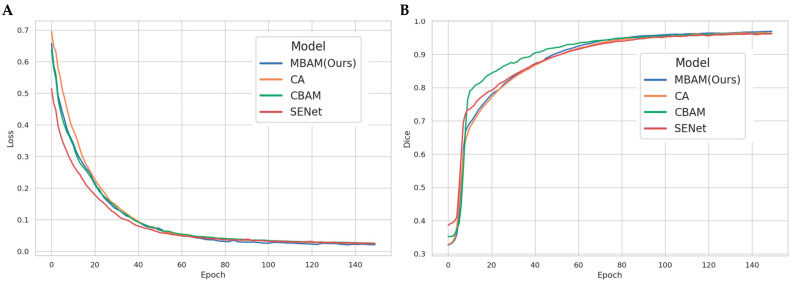
Training loss and validation *Dice* curves of different attention modules: (**A**) *Loss* over epochs. (**B**) *Dice* over epochs.

To verify the effectiveness of the proposed Moso Bamboo Multi-scale Attention Module (MBAM), comparative experiments were conducted with three widely used attention mechanisms, which are SENet (Squeeze-and-Excitation Network), CBAM (Convolutional Block Attention Module), and CA (Coordinate Attention). All attention modules were embedded into the same U-Net backbone and trained and tested under identical settings. From the training loss and validation *Dice* curves of different attention modules (Figure 6A), it can be observed that all models exhibit rapid and stable convergence during the early training stage. As the number of epochs increases, the loss curves gradually flatten and enter a stable phase after approximately 80 epochs. Among all models, MBAM achieves the lowest final loss value, highlighting its superior capability in suppressing noise and enhancing effective feature representations. In terms of validation of *Dice* improvement (Figure 6B), CBAM shows a relatively faster performance gain in the early stage and reaches a high *Dice* level earlier. However, as training progresses, the performance improvement of CBAM gradually saturates and fails to maintain its advantage in the final convergence stage. In contrast, the *Dice* curve of MBAM continues to rise steadily during later training stages.

Table 3 summarizes the segmentation performance of different attention modules on the validation set, including *Dice*, *IoU*, *Overall Accuracy*, and ${mAP}_{50-95}^{mask}$. The results show that MBAM achieves the best performance on all three pixel-level metrics (*Dice*, *IoU*, and *Overall Accuracy*). CA slightly outperforms others on ${mAP}_{50-95}^{mask}$, indicating that coordinate-guided attention is more sensitive to elongated structures under higher *IoU* thresholds. Considering overall pixel-level overlap metrics, MBAM demonstrates greater robustness with minimal additional computational cost, satisfying the design goal of boundary refinement required for moso bamboo cell segmentation.

From the quantitative results in Table 3, MBAM (Ours) achieves the best performance across all three core pixel-level metrics: *Dice* = 0.9690, *IoU* = 0.9324, and *Overall Accuracy* = 0.9707. Compared with CA, CBAM, and SENet, MBAM improves *IoU* by +0.0012, +0.0024, and +0.0043, respectively. *Dice* is further improved from the range of 0.9622–0.9640 to 0.9690, while *Overall Accuracy* also ranks first within the range of 0.9688–0.9707. For instance-level mask quality, CA achieves the highest ${mAP}_{50-95}^{mask}$ of 0.9314, whereas the remaining three methods all reach 0.9286, with differences on the order of 0.0028. These comparisons indicate that MBAM achieves comprehensive superiority in pixel-level overlap metrics (*Dice*/*IoU*/*Overall Accuracy*), while CA exhibits a slight advantage in cross-threshold instance matching (${mAP}_{50-95}^{mask}$). In terms of stability and performance margin, MBAM consistently leads across all three pixel-level metrics with balanced improvements: the maximum *IoU* gain reaches +0.0043 (vs. SENet), and the maximum *Dice* gain is approximately +0.0068 (vs. SENet), demonstrating systematic improvements in pixel-level overlap and overall accuracy. Although MBAM is slightly lower than CA in ${mAP}_{50-95}^{mask}$ by 0.0028, the difference is statistically small. Overall, MBAM and CA show complementary strengths across different evaluation dimensions, while the remaining methods fall into a secondary performance tier.

#### 3.1.2. Comparison of Segmentation Models

**Figure 7:**
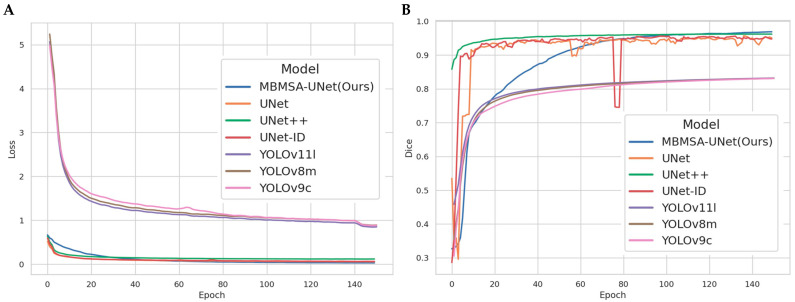
Training loss and validation *Dice* curves of different segmentation models: (**A**) *Loss* over epochs. (**B**) *Dice* over epochs.

To comprehensively evaluate the overall performance of MBMSA-UNet, several classical and lightweight instance segmentation models were selected as baselines, including U-Net, UNet++, U-Net-ID, YOLOv8m-seg, YOLOv9c-seg, YOLO11l-seg, and MBMSA-UNet (Ours). All models were trained on the same dataset with identical hyperparameter settings, and evaluation metrics and computational environments were kept consistent with those in Section 2 to ensure fairness and comparability. From the loss curves of different segmentation models (Figure 7A), it can be observed that U-Net-based models converge significantly faster than YOLO-based models, which is primarily attributed to differences in architectural design. Due to its increased structural complexity, MBMSA-UNet shows a slightly slower loss decrease in the early stage, but its overall curve is smoother and more stable. In contrast, YOLO-based models exhibit consistently higher loss values and slower convergence, which can be attributed to their detection-oriented architecture and multi-task loss formulation, making them less efficient for pixel-level optimization. The validation *Dice* curves (Figure 7B) further highlight the performance differences among the models. Although MBMSA-UNet shows a moderate growth rate in the early stage, its *Dice* curve continues to increase steadily as training progresses, eventually surpassing other U-Net variants and achieving the highest *Dice* score. The YOLO-based models maintain relatively low *Dice* values throughout training, stabilizing around 0.8, which is insufficient for fine-grained segmentation tasks. This further confirms the inherent limitations of general-purpose detection models in microscopic structure segmentation.

Table 4 summarizes the quantitative performance of different models. Compared with the standard U-Net, MBMSA-Unet achieves substantial improvements in *Dice*, *IoU*, and ${mAP}_{50–95}^{mask}$, significantly enhancing boundary consistency and reducing missed detections for adhesive cells. Compared with U-Net-ID and Unet++, MBMSA-Unet achieves higher *Dice* and *IoU* while maintaining a parameter count and computational cost close to U-Net-ID and far lower than Unet++ in terms of FLOPs, demonstrating a superior accuracy-efficiency trade-off. The YOLO series performs relatively poorly on this task, mainly due to its bias toward object-level detection and coarse-grained masks, which struggle to capture dense cell wall structures. This further validates the necessity of combining an encoder–decoder backbone with multi-scale attention mechanisms. As shown in Table 4, MBMSA-Unet achieves the best performance across all four core metrics: *Dice* = 0.9690, *IoU* = 0.9324, *Overall Accuracy* = 0.9707, and ${mAP}_{50–95}^{mask}$ = 0.9286. Compared with the baseline U-Net, the improvements are +2.65, +3.95, +1.54, and +5.43 percentage points, respectively, indicating significant advantages in both pixel-level overlap and instance matching. Compared with U-Net-ID and Unet++, MBMSA-Unet remains superior in *Dice* and *IoU*, and also achieves higher ${mAP}_{50–95}^{mask}$ (0.9286 vs. 0.9086/0.9014). The YOLOv8m-seg, YOLOv9c-seg, and YOLOv11l-seg models perform significantly worse across all metrics than U-Net-based methods. In terms of resource efficiency, MBMSA-Unet has approximately 26.37 M parameters and 43.19 B FLOPs, comparable to U-Net-ID (25.98 M/42.02 B). Compared with Unet++ (9.16 M/117.29 B), MBMSA-Unet achieves higher accuracy with substantially lower computational cost. Relative to U-Net (8.64 M/12.79 B), MBMSA-Unet incurs higher computation but yields substantial accuracy gains. Compared with YOLO-based models (~27–28 M/110–145 B), MBMSA-Unet requires significantly fewer FLOPs while achieving markedly higher accuracy. These results demonstrate that MBMSA-Unet achieves comprehensive superiority in *Dice*, *IoU*, *Overall Accuracy*, and ${mAP}_{50–95}^{mask}$ under resource consumption comparable to U-Net-ID, while significantly outperforming Unet++ and YOLO-based models in overall efficiency.

### 3.2. Visualization

#### 3.2.1. Visualization of Attention Modules

**Figure 8:**
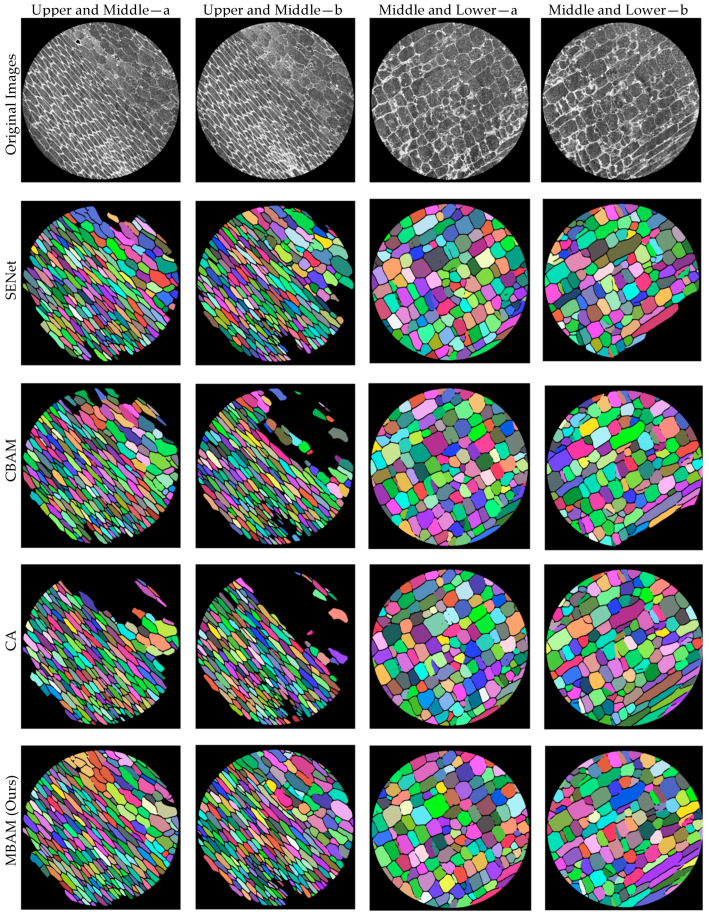
Segmentation results of different attention modules on moso bamboo cell images.

Four representative moso bamboo cell microscopic images were selected for visualization. Images Upper and Middle—a and Upper and Middle—b are from the upper-middle region, where cells are relatively elongated with weaker contrast; images Middle and Lower—a and Middle and Lower—b are from the lower-middle region, where cells tend to be polygonal with more regular boundaries. All images were processed using identical preprocessing and the same segmentation backbone to eliminate external interference and highlight the impact of attention mechanisms on feature representation and boundary modeling. Figure 8 adopts a 5 × 4 matrix layout. The first row shows the original microscopic images, while the second to fifth rows present segmentation results using SENet, CBAM, CA, and MBAM, respectively. Columns correspond to the same images to facilitate cross-module comparison, while rows correspond to the same module to assess robustness across different cell morphologies. Module names are indicated on the left, and image IDs are labeled at the top. Overall, the four attention mechanisms exhibit consistent differences in boundary continuity, separation of adhesive cells, and background suppression. SENet enhances major structures through channel recalibration but still shows local boundary breaks or jagged edges in elongated, low-contrast regions (upper- and middle-type samples). CBAM alleviates some boundary discontinuities via joint channel-spatial modeling, yet fragmented masks persist in regions with uneven illumination or local overexposure. CA, benefiting from coordinate information, is more sensitive to elongated structures and produces smoother contours in upper- and middle-type images but occasionally causes over-smoothing and slight boundary shrinkage in middle- and lower-type images with multi-directional edges. In contrast, MBAM demonstrates more stable fine boundaries and fewer spurious responses across both image types. In the upper- and middle-type images, adhesion regions form clearer low-response separation bands, effectively mitigating the coexistence of under- and over-segmentation. In the middle- and lower-type images, corner points and fine cracks are better preserved, while overexposed intracellular textures and background noise are more effectively suppressed. Considering both visual quality and pixel-level consistency, MBAM achieves the most balanced performance across all four samples, maintaining global coherence while refining fine boundaries.

#### 3.2.2. Visualization of Segmentation Models

**Figure 9:**
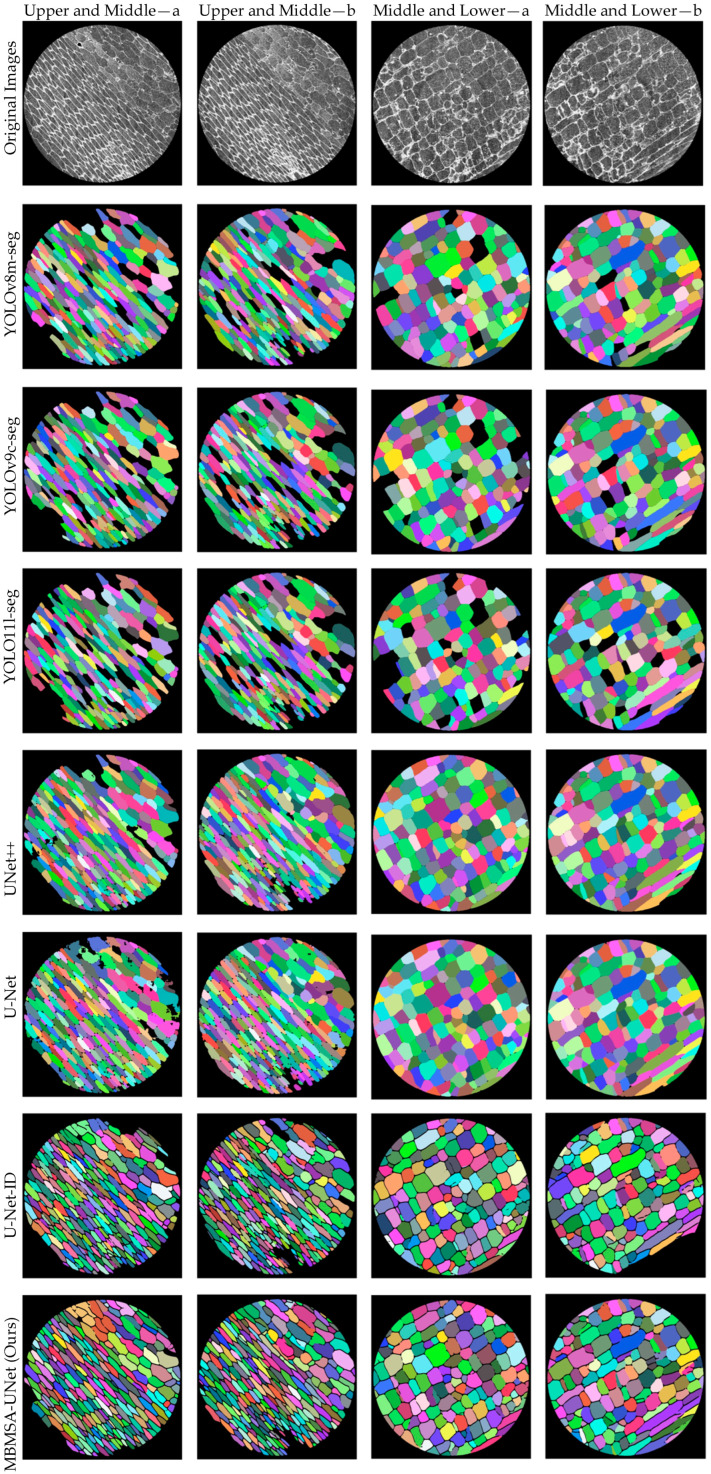
Segmentation results of different models on moso bamboo cell images.

Using the same four representative images, qualitative comparisons were conducted among seven segmentation models, as shown in Figure 9: YOLOv8m-seg, YOLOv9c-seg, YOLO11l-seg, UNet++, U-Net, U-Net-ID, and the proposed MBMSA-UNet.

All inference results were obtained under identical data and training configurations to ensure that observed differences primarily stem from the model architecture. The first row shows the original images, followed by the segmentation results of the seven models. Columns correspond to the four image types (Upper and Middle—a, Upper and Middle—b, Middle and Lower—a, and Middle and Lower—b), enabling horizontal comparison within the same image, while rows allow assessment of model robustness across different image types. The visualization results reveal significant differences among models in boundary refinement, region consistency, and weak-contrast recognition. The YOLO-based models (v8/v9/11-seg) tend to produce blocky masks and staircase-like edges in dense regions, with insufficient continuity of cell walls and frequent holes or deletions in weak-contrast areas. UNet++ generates relatively smooth boundaries but still exhibits texture-induced false segmentations in upper- and middle-type samples. U-Net shows poor stability in this task, with some missing or abnormal masks, indicating that basic semantic segmentation models are insufficient for this application. U-Net-ID produces more regular contours but still suffers from occasional connectivity issues in tightly packed boundaries. In contrast, MBMSA-UNet consistently produces finer and more continuous cell walls across all four images, with clearer separation between adjacent cells. It avoids both large merged under-segmentation and texture- or overexposure-induced holes. For weak-contrast upper- and middle-type cells, MBMSA-UNet maintains coherent boundary responses without excessive expansion; for polygonal B-type cells, it preserves corner points and fine cracks with better morphological consistency. Nevertheless, in scenarios with extremely uneven cell distribution or extremely narrow gaps, slight boundary shrinkage or corner over-smoothing may still occur. Future work may incorporate stronger boundary-aware losses or morphological consistency regularization to further improve the separability of sharp corners and micro-gaps while maintaining pixel-level accuracy and enhancing instance-level geometric fidelity. A representative failure case is analyzed in the following section.

#### 3.2.3. Failure Case Analysis

**Figure 10:**
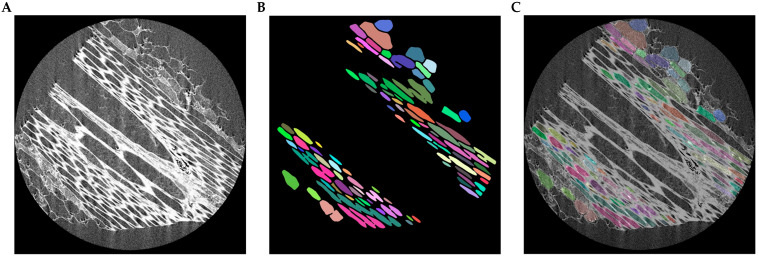
Failure case example of MBMSA-UNet on challenging bamboo cell structures: (**A**) Original image. (**B**) Predicted instance segmentation results. (**C**) Overlay visualization.

Although MBMSA-UNet achieves strong segmentation performance in most scenarios, several challenging cases still remain. Figure 10 presents a representative failure example. In this image, the fiber cells are extremely densely packed, and the intercellular gaps are extremely narrow. As a result, the boundaries between neighboring cells become weak or partially indistinguishable.

As shown in Figure 10B, several adjacent cells are predicted as a single connected region, leading to under-segmentation. The overlay visualization in Figure 10C further highlights that these errors mainly occur in regions where cell walls are extremely thin or locally blurred. In such cases, the boundary responses generated by the network are insufficient to fully separate adjacent cells. These observations indicate that the current model may still struggle with extremely narrow cell walls and highly compressed cellular arrangements. Future work could incorporate stronger boundary-aware losses or topology-preserving regularization to further improve the separation of densely adhered cells.

## 4. Discussion

This study focuses on three key challenges in microscopic images of moso bamboo cells—namely, densely packed cell walls, blurred boundaries, and local overexposure—and proposes MBMSA-UNet, which adopts the encoder–decoder architecture of U-Net as its backbone. Multi-scale channel-spatial joint attention modules (MBAM) are inserted at critical fusion locations in both the encoder and decoder. Experimental results and visual comparisons show that the cascaded modeling strategy of MBAM enhances responses in boundary and overexposed regions while suppressing pseudo-activations induced by overexposed textures, without significantly increasing computational cost. Pixel-level metrics (*Dice*, *IoU*, and *Overall Accuracy*) remain stable and consistently within the top-performing range across four representative sample types, while instance-level mask quality is maintained at a level comparable to the best-performing methods. These findings indicate that multi-scale channel recalibration and spatial saliency gating are particularly crucial for plant tissues such as moso bamboo, which exhibit large structural scale variations and long, fine boundaries.

Compared with object-level segmentation approaches in the YOLO family, encoder–decoder backbones exhibit inherent advantages in fine-grained boundary delineation within densely packed cellular regions. Relative to improved U-Net variants such as U-Net-ID and UNet++, the proposed method achieves better boundary continuity and global consistency while maintaining a comparable number of parameters, highlighting the effectiveness of the attention insertion points and information flow design.

These factors define the applicability limits of the proposed approach. Nevertheless, several aspects of the model remain open for improvement. First, under scenarios with extremely uneven illumination or ultra-narrow intercellular gaps, a small number of samples exhibit slight boundary shrinkage or excessive smoothing at sharp corners, suggesting that the current loss function does not yet impose sufficient constraints on geometric morphology. Second, although the model demonstrates good robustness on samples from both the upper-middle and lower-middle regions, the data distribution is still dominated by specific imaging conditions and sample sources; thus, cross-laboratory and cross-modality generalization requires more systematic validation. Third, instance-level segmentation still relies on pixel-wise probability maps followed by thresholding and matching strategies. In addition, factors such as sample tilt angles during scanning, external structural damage, and variations in cultivation conditions may also influence the imaging quality and structural characteristics of the samples, which may affect the performance of the proposed method in more complex scenarios. When confronted with highly compact cell clusters, further improving the separability and topological consistency of instance boundaries remains a challenge that calls for coordinated optimization between network architecture design and post-processing strategies.

Based on these observations, future work can be advanced in three directions. First, boundary-aware or morphology-preserving regularization terms—such as level-set or contour-based losses, distance-transform-based boundary losses, and topological consistency constraints—can be incorporated into the learning objectives to alleviate excessive smoothing at sharp corners and ultra-thin gaps. Second, structurally, joint designs that combine attention mechanisms with decoupled upsampling or multi-resolution pyramids can be explored, or lightweight boundary refinement branches can be introduced after skip connections to further suppress shallow-layer noise during decoding while improving alignment with high-level semantic features. Third, at the data and application levels, cross-modality and cross-scenario evaluations (e.g., bright-field, polarized light, confocal microscopy, and micro-CT slices) should be expanded. In addition, few-shot self-supervised or semi-supervised learning strategies, together with uncertainty estimation, can be integrated to enhance transferability and interpretability in real-world production workflows. Meanwhile, for edge-device deployment, knowledge distillation and structural sparsification can be investigated to further reduce latency and energy consumption while maintaining segmentation accuracy.

## 5. Conclusions

This study addresses the task of instance segmentation of moso bamboo cells and proposes a multi-scale attention-based model, termed MBMSA-UNet. The model introduces a plug-and-play MBAM module into the U-Net framework and adopts a cascaded strategy of channel recalibration followed by spatial saliency enhancement, jointly reinforcing the representation of cell boundaries and low-contrast regions at critical stages of both encoding and decoding. This design effectively suppresses mis-segmentation caused by tightly packed and blurred cell walls as well as local overexposure artifacts. Comprehensive quantitative evaluations and qualitative visualizations demonstrate that MBMSA-UNet achieves leading performance on pixel-level metrics such as *Dice*, *IoU*, and *Overall Accuracy* while maintaining competitive quality in instance-level mask segmentation. In terms of computational cost, the proposed model remains on par with mainstream improved U-Net variants and significantly outperforms detection-oriented segmentation frameworks. These results indicate that, for plant tissue images such as moso bamboo that exhibit pronounced structural scale variations and densely distributed fine boundaries, the synergy between encoder–decoder backbones and multi-scale attention mechanisms provides an effective balance between segmentation accuracy and computational efficiency. Future work will focus on three directions: boundary- and morphology-aware learning, cross-modality generalization, and efficient deployment strategies, with the aim of further improving the robustness and accuracy of cell segmentation under complex microscopic imaging conditions.

## Figures and Tables

**Table 1:** Experimental environment configuration.

Categories	Configuration
CPU	AMD EPYC 9K84 96-Core Processor (128 vCPUs)
GPU	NVIDIA H20
Operating system	Ubuntu Linux (64-bit)
Programming language	R 4.x + Python 3.8
Framework	TensorFlow 2.x/PyTorch 2.4.1 + CUDA 12.x + cuDNN 9.x

**Table 2:** Key training parameters.

Training Parameters	Values
Input image size	256 × 256 (RGB)
Epochs	150
Batch size	12
Initial learning rate	1.0 × 10^−4^
Learning rate decay	1.0 × 10^−15^
Optimizer	RMSprop

**Table 3:** Comparison of different attention modules.

Modules	*Dice*	*IoU*	*Overall Accuracy*	${\mathrm{mAP}}_{50-95}^{\mathrm{mask}}$
SENet	0.9622	0.9281	0.9688	0.9286
CBAM	0.9634	0.9300	0.9698	0.9286
CA	0.9640	0.9312	0.9695	0.9314
MBAM (Ours)	0.9690	0.9324	0.9707	0.9286

**Table 4:** Comparison of different segmentation models.

Models	*Dice*	*IoU*	*Overall Accuracy*	${\mathrm{mAP}}_{50–95}^{\mathrm{mask}}$	*Params* (*M*)	*FLOPs* (*B*)
U-Net	0.9425	0.8929	0.9553	0.8743	8.64	12.79
Unet++	0.9623	0.9279	0.9653	0.9014	9.16	117.29
U-Net-ID	0.9564	0.9176	0.9655	0.9086	25.98	42.02
YOLOv8m-seg	0.7980	0.6805	0.9297	0.5573	27.3	110.2
YOLOv9c-seg	0.7823	0.6586	0.9270	0.5455	27.4	145.5
YOLOv11l-seg	0.8034	0.6864	0.9312	0.5724	27.6	132.2
MBMSA-Unet (Ours)	0.9690	0.9324	0.9707	0.9286	26.37	43.19

## Data Availability

The microscopic CT image datasets generated and analyzed during the current study are not publicly available due to privacy and proprietary restrictions, but they are available from the corresponding authors upon reasonable request.
